# Autophagy Inhibition Enhances Anti-Glioblastoma Effects of Pyrazolo[3,4-*d*]pyrimidine Tyrosine Kinase Inhibitors

**DOI:** 10.3390/life12101503

**Published:** 2022-09-27

**Authors:** Sofija Jovanović Stojanov, Ana Kostić, Mila Ljujić, Ema Lupšić, Silvia Schenone, Milica Pešić, Jelena Dinić

**Affiliations:** 1 Department of Neurobiology, Institute for Biological Research “Siniša Stanković”—National Institute of the Republic of Serbia, University of Belgrade, Bulevar Despota Stefana 142, 11060 Belgrade, Serbia; 2 Institute of Molecular Genetics and Genetic Engineering (IMGGE), University of Belgrade, Vojvode Stepe 444a, 11042 Belgrade, Serbia; 3 Department of Pharmacy, University of Genova, Viale Benedetto XV 3, 16132 Genova, Italy

**Keywords:** glioblastoma, autophagy, Src tyrosine kinase inhibitor, multidrug resistance

## Abstract

Drug resistance presents a major obstacle to the successful treatment of glioblastoma. Autophagy plays a key role in drug resistance, particularly in relation to targeted therapy, which has prompted the use of autophagy inhibitors to increase the effectiveness of targeted therapeutics. The ability of two Src tyrosine kinase inhibitors, Si306 and its prodrug pro-Si306, to induce autophagy was evaluated in the human glioblastoma cell line U87 and its multidrug-resistant counterpart U87-TxR. Autophagy markers were assessed by flow cytometry, microscopy, and Western blot, and induction of autophagy by these compounds was demonstrated after 3 h as well as 48 h. The effects of Si306 and pro-Si306 on cell proliferation and cell death were examined in the presence or absence of autophagy inhibition by bafilomycin A1. Combined treatments of Si306 and pro-Si306 with bafilomycin A1 were synergistic in nature, and the inhibition of autophagy sensitized glioblastoma cells to Src tyrosine kinase inhibitors. Si306 and pro-Si306 more strongly inhibited cell proliferation and triggered necrosis in combination with bafilomycin A1. Our findings suggest that modulation of Si306- and pro-Si306-induced autophagy can be used to enhance the anticancer effects of these Src tyrosine kinase inhibitors and overcome the drug-resistant phenotype in glioblastoma cells.

## 1. Introduction

Autophagy is a self-digestive pathway that maintains cellular homeostasis through recycling intracellular components and the elimination of damaged organelles and unnecessary proteins [1]. This is an evolutionarily conserved process in eukaryotes and occurs at a basal rate in all cells. Defects in autophagy have been shown to correlate with the propensity of various diseases, including cancer. In normal tissue, autophagy plays a major housekeeping role in preventing tumorigenesis by eliminating damaged organelles and alleviating oxidative stress, thereby executing a cytoprotective role and providing genome stability [2,3]. However, once the primary tumor has been established, this system is hijacked by tumor cells. Proliferating cancer cells have elevated demands for metabolites, and in order to survive, component recycling through autophagy compensates for nutrient deficiency and helps cancer cells adapt to a hypoxic environment [2]. In cancer, autophagy can play a dual role that is context-dependent and can be both cytoprotective and cytotoxic [2]. Since it is rapidly upregulated under cellular stress conditions, autophagy can provide an alternative source of energy for cancer cell survival, especially in cancer cells exposed to anticancer treatment [4]. Autophagy may increase cellular metabolism, which inactivates drugs and supports the development of drug resistance [3]. In fact, raising the level of autophagy is one of the key mechanisms by which cancer cells acquire resistance to anticancer agents. However, excessive and/or prolonged autophagy induced by therapeutic stress may also lead to caspase-independent cell death [3]. Thus, autophagy induction as a therapeutic option can cause varying cellular responses and largely depends on the treatment and type of tumor, as well as the stage of the disease [3].

Drug resistance represents a major obstacle to successful cancer treatment. Glioblastoma, the most common and most aggressive malignant tumor of the central nervous system, is characterized by a high rate of cell proliferation, infiltrating nature, and resistance to therapy [5]. The resistance of glioblastoma to various therapies is largely a result of an exceptionally mutated genome and increased activity of tyrosine kinase receptors, such as EGFR, which is typically upregulated in glioblastoma [6]. In addition to innate resistance, glioblastoma cells may also develop a resistant phenotype in response to therapy. In both cases, the expression of different genes is altered, leading to multidrug resistance (MDR) and a reduced response of cancer cells to treatment [7]. Glioblastoma resistance has been shown for a wide range of chemotherapeutic agents including temozolomide, paclitaxel, etoposide, vincristine, carboplatin, and irinotecan [8]. 

Autophagy is currently considered a vital element of drug resistance in a variety of non-solid and solid tumors, including glioblastoma [9]. As a result, the main research focus has been on controlling cell growth, cell death, and drug resistance by modulating autophagy. Changing autophagy levels or inhibiting the expression of autophagy-related genes has been shown to improve drug efficacy and help overcome autophagy-induced drug resistance [9]. Currently, the clinical focus is on the use of pharmacological inhibitors of autophagy in combination with standard chemotherapy or targeted therapy, as numerous studies have shown that such combinations can increase cell death in drug-resistant cancer cells [2,10,11,12].

Tyrosine kinases are a family of enzymes that phosphorylate tyrosine residues of specific proteins. In solid tumors, both receptor and non-receptor tyrosine kinases are overexpressed and play an important role in cancer development [13]. Src tyrosine kinase belongs to the largest family of non-receptor tyrosine kinases, the Src family tyrosine kinases (SFKs), and is their most widely characterized member. The deregulation of the activity of SFKs is accountable for the growth and progression of various types of cancer [4], while particularly elevated Src activity has been reported in glioblastoma [14]. For this reason, Src represents a target for cancers where this kinase is involved and/or overexpressed. Many anticancer therapies are based on the use of tyrosine kinase inhibitors. These inhibitors are small molecules with hydrophobic properties that readily penetrate cells and inhibit both cytoplasmic and membrane tyrosine kinases and regulate signaling pathways involved in cell survival, proliferation, and differentiation [15].

Si306 and its prodrug pro-Si306 are novel small-molecule compounds and ATP-competitive inhibitors of Src tyrosine kinase and other SFK members. They are based on the pyrazolo[3,4-*d*]pyrimidine scaffold [16,17,18] and are capable of passing through the blood–brain barrier [19]. The strong activity of Si306 and pro-Si306 against different types of cancer, including glioblastoma, was reported in numerous in vitro and in vivo studies [17,18,19,20,21]. We have previously shown that Si306 and its prodrug act as dual-targeting molecules with the ability to inhibit both SFKs and P-glycoprotein, an efflux transporter overexpressed on the membrane of multidrug-resistant glioblastoma cells [20]. These Src tyrosine kinase inhibitors (STKIs) also considerably reduced the invasive properties of several patient-derived glioblastoma cultures, as well as glioblastoma xenografts in the zebrafish embryo model [19]. In addition, Si306 and pro-Si306 showed strong antiproliferative and pro-oxidative potential in primary glioblastoma cells leading to senescence and necrosis [18]. Often, the biological activity of prodrugs is significantly lower compared with the parent drug [22]. However, our previous studies demonstrated that pro-Si306 showed similar biological activity compared to Si306, while also displaying improved pharmacological properties [23].

These encouraging results have prompted further research into these Src tyrosine kinase inhibitors as potential targeted therapeutics in the treatment of glioblastoma. This study aimed to evaluate the effect of Si306 and pro-Si306 on autophagy in U87 and MDR U87-TxR glioblastoma cells and whether the modulation of autophagy can improve the anticancer activity of these compounds and help overcome MDR.

## 2. Materials and Methods

### 2.1. Drugs

Si306 and pro-Si306 were obtained as previously described [23]. Bafilomycin A1 (Baf A1) was acquired from Sigma-Aldrich Chemie GmbH (Taufkirchen, Germany). Si306 and pro-Si306 were stored at room temperature (RT) as 20 mM aliquots in dimethyl sulfoxide (DMSO). Baf A1 was stored at 4 °C as 20 µM aliquots in DMSO. Sterile Milli-Q water was used to dilute all drugs directly before treatment.

### 2.2. Reagents

Minimum Essential Medium (MEM) was acquired from Capricorn Scientific GmbH (Ebsdorfergrund, Germany). L-glutamine, trypsin, and penicillin–streptomycin solution were from Biowest (Nuaillé, France). Thiazolyl blue tetrazolium bromide (MTT), fetal bovine serum (FBS), DMSO, and Acridine orange (AO) were obtained from Sigma-Aldrich Chemie GmbH. Hoechst 33342 and carboxyfluorescein succinimidyl ester (CFSE) were purchased from Thermo Fisher Scientific (Waltham, MA, USA). An Apoptosis Detection Kit based on Annexin-V-FITC (AV) and propidium iodide (PI) staining was acquired from Abcam (Cambridge, UK). Triton™ X-100 was purchased from Merck KGaA (Darmstadt, Germany), while bovine serum albumin (BSA) was obtained from Serva (Heidelberg, Germany). Anti-LC3A/B rabbit primary antibody was from Cell Signaling Technology^®^ (Danvers, MA, USA) and p62/SQSTM1 mouse primary antibody was purchased from Novus Biologicals (Littleton, CO, USA). Secondary antibodies Alexa Fluor^®^ 488 goat anti-mouse IgG (H + L) and Alexa Fluor^®^ 555 goat anti-rabbit IgG (H + L) were obtained from Thermo Fisher Scientific.

### 2.3. Cell Cultures

Human glioblastoma cell line U87 was acquired from American Type Culture Collection (Rockville, MD, USA). Multidrug-resistant U87-TxR cell line was selected from parental U87 cell line that was continuously exposed to increasing concentrations of paclitaxel [24]. U87 and U87-TxR cells were maintained in MEM supplemented with 10% FBS, 2 mM L-glutamine, 10,000 U/mL penicillin, and 10 mg/mL streptomycin. Cells were cultured at 37 °C in a humidified atmosphere containing 5% CO_2_.

### 2.4. MTT Assay

U87 and U87-TxR cells were seeded into 96-well tissue culture plates (4000 cells/well) and allowed to attach overnight at 37 °C. Cells were then treated for 48 h with Si306 and pro-Si306 (1, 2.5, 5, 10, and 25 µM), as well as Baf A1 (10, 20, and 50 nM) to assess cell growth. U87 and U87-TxR cells cultured in MEM alone were used as a negative control, while 0.25% (*v*/*v*) DMSO served as a solvent control. Baf A1 is a well-known vacuolar-type H(+)-ATPase inhibitor that blocks autophagosome–lysosome fusion [25,26]. In addition, increasing concentrations of Si306 and pro-Si306 (5, 10, and 25 μM) were applied with 20 nM Baf A1 in simultaneous treatments. This colorimetric assay is based on enzymatic reduction in the MTT into formazan dye by active mitochondria in viable cells, indicating their metabolic activity. Following the treatment, 100 μL of MTT solution (2 mg/mL) was added to each well, and the plates were incubated at 37 °C for 4 h. The formazan crystals formed in cells with viable mitochondria were dissolved in 200 µL of DMSO, and the absorbance was measured at 570 nm in an automated microplate reader (Thermo Scientific™ Multiskan™ Sky Microplate Spectrophotometer, Waltham, MA, USA).

### 2.5. Median Effect Analysis

The combined effects of Si306 or pro-Si306 and Baf A1 were evaluated in U87 and U87-TxR cells. Briefly, the cells were trypsinized, seeded into 96-well tissue culture plates at 4000 cells/well, and incubated overnight at 37 °C. In single treatments, the cells were treated for 48 h with increasing concentrations of Si306 or pro-Si306 (5, 10, and 25 µM) or increasing concentrations of Baf A1 (10, 20, and 50 nM). In simultaneous treatments, 20 nM Baf A1 was combined with increasing concentrations of Si306 or pro-Si306. Following the treatments, the cell growth inhibition was analyzed by MTT assay as described. The absorbance was measured at 570 nm in an automated microplate reader (Thermo Scientific™ Multiskan™ Sky Microplate Spectrophotometer, Waltham, MA, USA). IC_50_ values were calculated by non-linear regression analysis using GraphPad Prism 6.0 (GraphPad Software, La Jolla, CA, USA).

The nature of the interaction between Si306 or pro-Si306 and Baf A1 was analyzed with CalcuSyn software v1.1 (Biosoft, Cambridge, UK) that uses the Combination Index (CI) method based on the multiple-drug-effect equation [27]. To evaluate the effect of both drugs in combination, the non-constant ratio was selected. The obtained results are shown in a fraction-affected CI graph. CI values < 1 point to synergism, with lower values indicating a greater degree of synergy. A CI value of 1 indicates an additive effect, while a CI value of >1 indicates an antagonistic effect.

### 2.6. Cell Death Detection by Flow Cytometry

The percentages of apoptotic, necrotic, and viable cells were assessed by flow cytometry using Abcam Apoptosis Detection Kit (AV/PI staining) according to the manufacturer’s instructions. U87 and U87-TxR cells were seeded into 6-well tissue culture plates (200,000 cells/well) and allowed to attach overnight at 37 °C. The next day, cells were treated for 48 h with 10 µM Si306 and pro-Si306, 20 nM Baf A1, or their combinations. After collection of adherent and detached cells by centrifugation, AV/PI staining was performed. The fluorescence intensity of AV/PI was immediately measured on a CyFlow Space flow cytometer (Partec, Münster, Germany) in FL1 and FL2 channels. At least 20,000 events were recorded per each sample, and Summit 4.3 software (Dako Colorado Inc., Fort Collins, CO, USA) was used to analyze the percentages of viable (AV− PI−), early apoptotic (AV+ PI−), late apoptotic (AV+ PI+), and necrotic (AV− PI+) cells.

### 2.7. CFSE Cell Proliferation Assay

CFSE staining was performed to assess cell proliferation by flow cytometry. The fluorescence intensity of CFSE progressively declines during cell divisions, thus allowing the assessment of cell proliferation rates. U87 and U87-TxR cells were trypsinized and stained with 5 μM CFSE for 15 min in the dark at 37 °C in 5% CO_2_. After incubation, cells were washed in PBS, seeded into 6-well tissue culture plates at a density of 200,000 cells/well and allowed to attach overnight at 37 °C. Following 48 h treatment with 10 µM Si306 and pro-Si306, 20 nM bafilomycin A1 or their combinations, cells were trypsinized, washed, and resuspended in PBS. Fluorescence intensity of CFSE dye was measured in FL1 channel on a CyFlow Space flow cytometer. At least 20,000 events were recorded per each sample, and the results were analyzed in Summit 4.3 software.

### 2.8. Western Blot

The protein levels of LC3 and p62 were evaluated by Western blot. U87 and U87-TxR cells were seeded into tissue culture flasks at 1 × 10^6^ cells per flask and allowed to attach overnight at 37 °C. The cells were then treated for 48 h with 10 µM Si306 or pro-Si306, 20 nM bafilomycin A1, or their combinations. After treatment, cells were directly lysed in Laemmli buffer (glycerol, 1M TRIS pH 6.8, 1% SDS, mQH2O, and 20% β-mercaptoethanol) with bromphenol blue, and proteins were loaded on 12% SDS-PAGE gels and separated by gel electrophoresis. Proteins were then transferred to PVDF membrane (Immobilon^®^-PSQ, Merck Millipore, Dublin, Ireland), and membranes were blocked in 5% non-fat dry milk (GE Healthcare, Buckinghamshire, UK) in TBST for 1 h at RT. Membranes were then incubated overnight at 4 °C with the following primary antibodies: rabbit polyclonal anti-LC3A/B (Cell Signaling; 4108) and mouse monoclonal anti-p62/SQSTM1 (Novus Biologicals; NBP2-43663), followed by Horse Radish Peroxidase (HRP)-conjugated anti-rabbit secondary antibody (Abcam, Cambridge, UK; ab6721) and anti-mouse secondary antibody (Dako; P0260) for 1 h at RT. Immunoreactivity was detected by iBright™ CL1500 Western Blot Imaging System (Thermo Fisher Scientific, Waltham, MA, USA). Each blot was reprobed with rabbit polyclonal anti-β-actin antibody (Abcam, Cambridge, UK; ab8227) and incubated with (HRP)-conjugated anti-rabbit secondary antibody. Densitometric analysis of immunoreactive bands was performed using ImageJ software (U.S. National Institutes of Health, Bethesda, MD, USA), and the results are expressed as relative values (i.e., density ratio normalized to the corresponding internal control, β-actin signal).

### 2.9. Transfection

U87 and U87-TxR cells were seeded into a 12-well tissue culture plate at 1.5 × 10^5^ cells/well in 500 µL of growth medium without penicillin/streptomycin and incubated overnight at 37 °C. On the following day, cells were transiently transfected with 0.5 μg of mRFP-LC3 plasmid using Lipofectamine 3000 (Thermo Fisher Scientific) according to the manufacturer’s protocol. The mRFP-LC3 plasmid was a gift from T. Yoshimori (Addgene plasmid #21075; http://n2t.net/addgene:21075, accessed on 16 September 2021; RRID:Addgene_21075) [28]. Cells were treated 24 h after transfection.

### 2.10. mRFP-LC3 Detection by Flow Cytometry and Fluorescence Microscopy

Extraction of cytoplasmic unbound fluorescently tagged LC3 with a mild detergent results in the fluorescence intensity that is directly correlated with autophagosome-bound LC3 and can be a good indicator of autophagic activity [29]. Transfected U87 and U87-TxR cells were trypsinized, centrifuged, and resuspended in 500 μL of growth medium. Cells were then treated with 10 μM Si306 or pro-Si306. After 3 h, cells were incubated with 0.05% saponin in PBS for 15 min. Following mild permeabilization, cells were washed and resuspended in 1 mL of PBS. The florescence intensity of mRFP-LC3 was immediately measured on a CyFlow Space flow cytometer in FL2 channel. At least 10,000 events were recorded per each sample, and the acquired results were analyzed in Summit 4.3 software. Subtraction of signal intensity between untreated and treated samples was performed and presented as overall difference in mRFP-LC3-positive cells.

To observe autophagosome formation, mRFP-LC3-transfected cells were seeded into 8-well chamber slides (Nunc, Nalgene, Denmark) at a density of 40,000 cells/chamber and treated with 10 μM Si306 or pro-Si306 for 3 h. After washing in PBS, cells underwent 4% paraformaldehyde fixation for 15 min at RT. The nuclei were labeled with Hoechst 33,342 for 15 min at RT, and the slides were mounted in Mowiol (Sigma-Aldrich Chemie GmbH, Taufkirchen, Germany). The cells were imaged under a Zeiss Axiovert inverted fluorescent microscope at 20× magnification (Carl Zeiss, Jena, Germany) using AxioVision 4.8 software.

### 2.11. Immunocytochemistry

For immunostaining, U87 and U87-TxR cells were seeded into 8-well chamber slides (Nunc, Nalgene, Denmark) at a density of 40,000 cells/chamber and allowed to attach overnight at 37 °C. The cells were then treated for 48 h with 10 µM Si306 or pro-Si306, 20 nM Baf A1, or their combinations. After washing in PBS, cells underwent 4% paraformaldehyde fixation for 15 min at RT and blocking in 0.5% BSA in PBS for 1 h. The cells were then incubated overnight at 4 °C with rabbit anti-LC3A/B and mouse anti-p62/SQSTM1 primary antibodies (1:1000 dilution in PBS/0.3% Triton X-100). On the following day, cells were washed in PBS, and fluorescent secondary antibodies Alexa Fluor 555 anti-rabbit IgG (H + L) and Alexa Fluor 488 anti-mouse IgG (H + L) were applied for 1 h at RT (1:1000 dilution in 0.5% BSA in PBS). The cells were stained with Hoechst 33,342 for 15 min at RT to label the nuclei and then mounted in Mowiol. The cells were imaged under a Zeiss Axiovert inverted fluorescent microscope at 20× magnification using AxioVision 4.8 software. LC3 puncta and p62 fluorescence intensity were quantified using ImageJ software. LC3-positive particles were quantified by counting the maxima. The corrected total cell fluorescence (CTCF) corresponding to p62 signal was calculated using the following formula: CTCF = Integrated density − (Area × Mean fluorescence of background readings).

### 2.12. Acridine Orange Assay

The autophagolysosomes were visualized by AO staining. After 48 h single treatments with 10 µM Si306 and pro-Si306 or co-treatments with 20 nM Baf A1, cells were washed in PBS and incubated for 15 min at 37 °C with 1 µM AO. Acridine orange accumulated in acidic compartments such as autophagolysosomes displays red fluorescence, while AO in cytoplasm and nuclei shows green fluorescence. After washing in PBS, cells were imaged live under Zeiss Axiovert inverted fluorescent microscope at 10× magnification using AxioVision 4.8 software.

### 2.13. Statistical Analysis

Statistical analysis was performed by GraphPad Prism 6.0 software. The Student’s *t*-test was used for two-group comparisons, and one-way analysis of variance (ANOVA) was used for comparing multiple groups. The accepted level of significance was *p* < 0.05.

## 3. Results

### 3.1. Si306 and Pro-Si306 Induce Autophagy in Glioblastoma Cells

**Figure 1:**
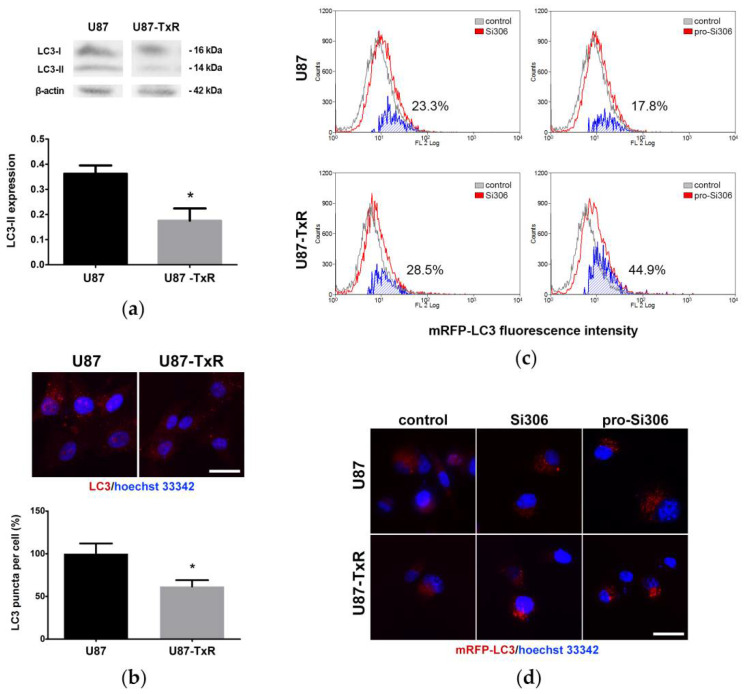
Si306 and pro-Si306 induce autophagy in glioblastoma cells. (**a**) Difference in expression levels of the autophagosomal marker LC3-II between U87 and U87-TxR cells. Representative Western blot images of LC3 protein expression in U87 and U87-TxR cells are shown. Histogram represents LC3-II expression normalized to β-actin. Values are presented as mean ± SEM (*n* = 3). Statistically significant difference between groups is shown as * (*p* < 0.05). (**b**) Fluorescence micrographs of autophagosomes in anti-LC3-labeled U87 and U87-TxR cells. Scale bar = 50 µm. Histogram shows LC3-positive puncta number in U87-TxR cells relative to U87 cells. Values are presented as mean ± SEM (*n* = 5). Statistically significant difference between groups is shown as * (*p* < 0.05). (**c**) Representative flow-cytometric profiles of mRFP-LC3 fluorescence intensity in transfected U87 and U87-TxR cells after 3 h treatment with Si306 or pro-Si306. Mild cell permeabilization was performed to remove the cytoplasmic unbound mRFP-LC3 and detect mRFP-LC3 bound to autophagic vacuoles. The percentages indicate an overall increase in the number of mRFP-LC3-positive cells (blue). (**d**) Fluorescence micrographs of autophagosomes in mRFP-LC3-transfected U87 and U87-TxR cells after 3 h treatment with Si306 or pro-Si306. Scale bar = 50 µm.

To compare the basal levels of autophagy in U87 and MDR U87-TxR cells, we evaluated the expression of the key autophagy marker LC3. As determined by immunoblotting, the levels of autophagosome membrane-bound LC3-II were two-fold higher in U87 cells compared with their resistant counterparts (Figure 1a). While fluorescence microscopy revealed that both cell lines have the ability to form autophagosomes, higher LC3-II levels in the parental cell line were also accompanied by a greater number of autophagosomes compared with MDR cells (Figure 1b). The number of LC3 puncta in the cytoplasm was approximately 40% higher in U87 cells, which may indicate differences in basal autophagic activity.

We then aimed to determine whether Src tyrosine kinase inhibitors Si306 and pro-Si306 can modulate autophagy in U87 and U87-TxR cells. Cells transfected with mRFP-LC3 were treated with Si306 and pro-Si306 for 3 h, and mRFP-LC3 fluorescence was measured by flow cytometry. As shown in Figure 1c, permeabilized cells showed more red florescence after treatment with STKIs in both cell lines, indicating that more LC3 was bound to autophagosomes as a result of increased autophagy. In addition, the formation of autophagosomes was observed in the cytoplasm after treatment with STKIs in both cell lines (Figure 1d). Si306 and its prodrug showed similar efficacy in inducing autophagic response in mRFP-LC3-transfected parental and MDR glioblastoma cells.

### 3.2. Si306 and Pro-Si306 Have Synergistic Interaction with Bafilomycin A1 in Glioblastoma Cells

**Figure 2:**
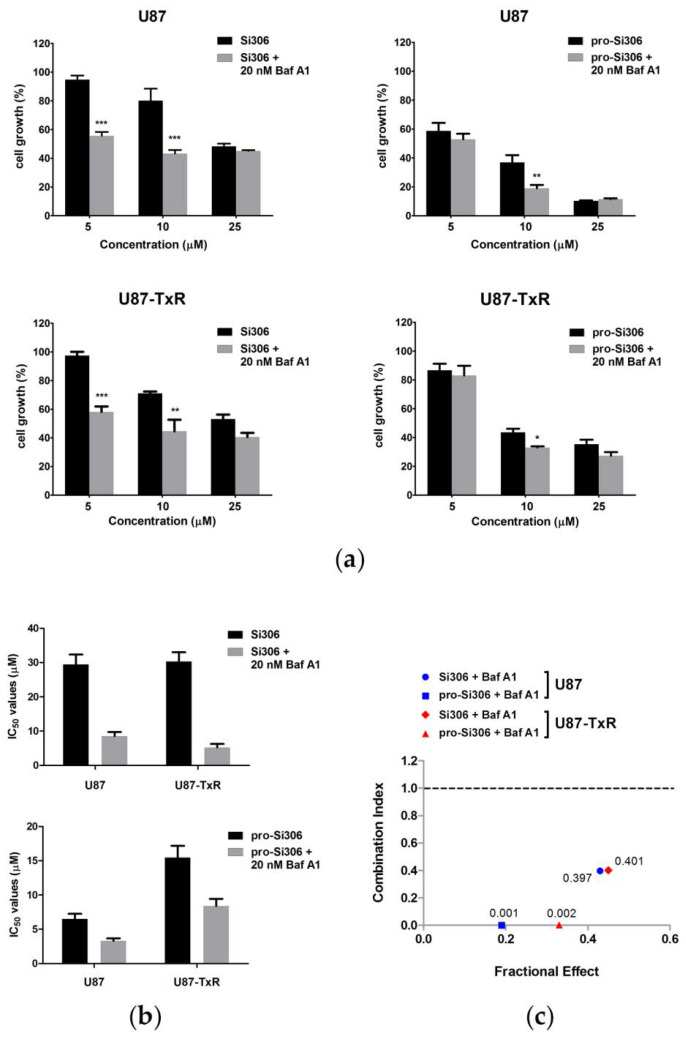
Si306 and pro-Si306 in combination with bafilomycin A1 (Baf A1) have a synergistic effect on growth inhibition of glioblastoma cells. (**a**) The sensitivity of U87 and U87-TxR cells to Si306 or pro-Si306 in single and combined treatments with Baf A1. The cell growth inhibition was assessed after 48 h by the MTT assay. The growth inhibitory effects of single Src tyrosine kinase inhibitor (STKI) treatments (black) were compared with combination treatments with Baf A1 (gray) in U87 and U87-TxR cells. Values are presented as mean ± SD (*n* = 3). Statistically significant difference between groups is shown as * (*p* < 0.05), ** (*p* < 0.01), and *** (*p* < 0.001). (**b**) IC_50_ values of Si306 and pro-Si306 in single and combination treatments with Baf A1. (**c**) Si306 and pro-Si306 have a synergistic interaction with Baf A1 in U87 and U87-TxR cells. The interactions between Si306 or pro-Si306 (10 µM) and Baf A1 (20 nM) in U87 and U87-TxR cells were analyzed using CalcuSyn software and presented as an algebraic estimate for each combination. Combination Index (CI) values lower than 1 indicate a synergistic interaction.

Since increased autophagy was observed after treatment with Si306 and pro-Si306, we evaluated the effect of the autophagic flux inhibitor Baf A1 on the efficacy of STKIs in U87 and U87-TxR cells after 48 h. The combined effects of STKIs and Baf A1 on cell growth inhibition were determined by the MTT assay (Figure 2a). The addition of a non-cytotoxic concentration of Baf A1 (20 nM) (Figure S1) improved the efficacy of Si306 and its prodrug in parental and MDR glioblastoma cells. This lysosomal inhibitor sensitized both cell lines to STKIs and significantly decreased the IC_50_ values of these inhibitors (Figure 2b). Specifically, the IC_50_ values of Si306 were reduced over three-fold and five-fold in U87 and U87-TxR cells, respectively. The IC_50_ values of the prodrug were reduced approximately two-fold in both cell lines. The most efficient sensitization was achieved by combining Baf A1 with 10 µM Si306 or pro-Si306 in both cell lines, so these concentrations were selected for further treatments.

The analysis of the nature of the interaction between compounds revealed a pronounced synergistic effect of the combined treatments with STKIs and Baf A1, with CI values below 1 in both glioblastoma cell lines (Figure 2c). The combination of 10 µM Si306 and 20 nM Baf A1 produced CI values of 0.397 in U87 and 0.401 in U87-TxR, respectively, indicating a synergistic interaction (Figure 2c). Similarly, CI values obtained after co-treatment with 10 µM pro-Si306 and 20 nM Baf A1 were 0.001 in U87 and 0.002 in U87-TxR, respectively. The synergy effects for multiple drug concentration combinations, expressed as CI values, are given in the Supplementary Material (Figure S2).

### 3.3. Si306 and Pro-Si306 Have Prolonged Effects on Autophagy in Glioblastoma Cells

**Figure 3:**
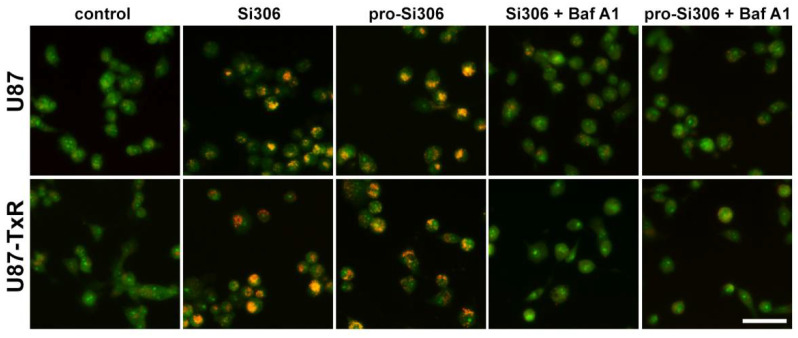
Acridine orange staining of glioblastoma cells. Fluorescence micrographs of U87 and U87-TxR cells after 48 h treatment with Si306 and pro-Si306 or their combination with Baf A1. U87 and U87-TxR cells treated with Si306 and pro-Si306 showed an acidified vacuolar staining that was absent in cells co-treated with Baf A1. Scale bar = 100 µm.

Considering the synergistic interaction between STKIs and Baf A1, we then assessed whether STKIs retained their effect on the autophagy induction after prolonged treatment using the acridine orange assay. After 48 h, the effect of Si306 and pro-Si306 on autophagy induction in U87 and U87-TxR cells was still evident, as visualized by autophagolysosome accumulation (Figure 3). Co-treatment with 20 nM Baf A1 resulted in the inhibition of lysosome acidification, indicating that autophagic flux was disrupted during the synergistic combination treatment (Figure 3).

**Figure 4:**
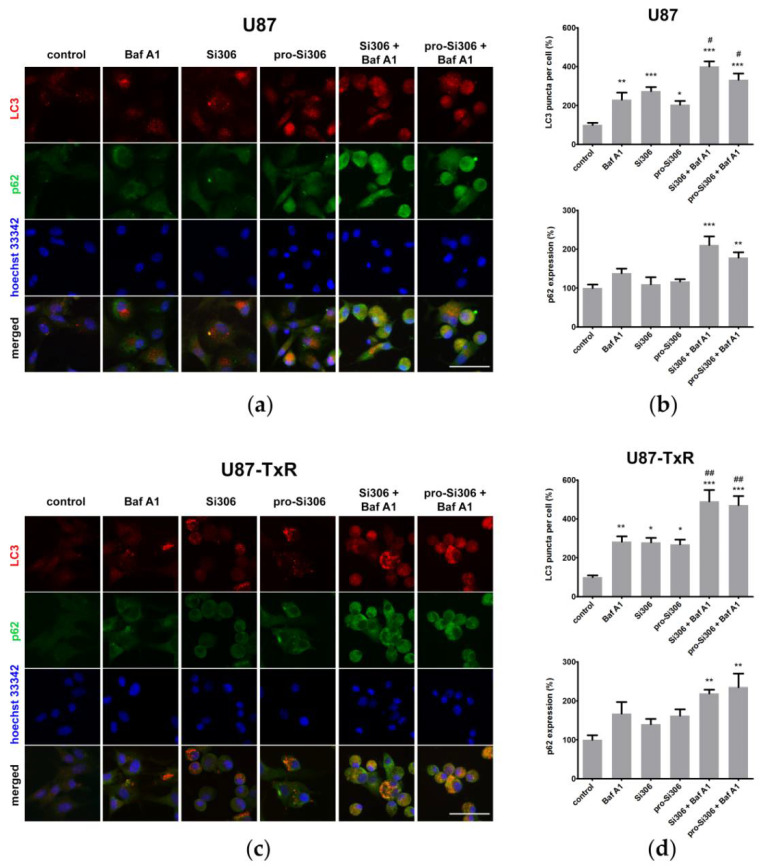
The effect of Si306 and pro-Si306 on autophagosome accumulation in glioblastoma cells in single and combined treatments with Baf A1. (**a**) Fluorescence micrographs of anti-LC3-labeled autophagosomes and p62 in U87 cells after 48 h treatment with Si306 and pro-Si306 or their combination with Baf A1. Nuclei were counterstained with Hoechst 33342. Scale bar = 100 μm. (**b**) Histograms show LC3-positive puncta number and anti-p62 fluorescence intensity (arbitrary units) in treated U87 cells normalized to control U87 cells and expressed as percentages. Values are presented as mean ± SEM (*n* = 4). Statistically significant difference between treated and control groups is shown as * (*p* < 0.05), ** (*p* < 0.01), and *** (*p* < 0.001). Statistically significant difference between single STKI treatment and combined treatment with Baf A1 is shown as # (*p* < 0.05). (**c**) Fluorescence micrographs of anti-LC3-labeled autophagosomes and p62 in U87-TxR cells after 48 h treatment with Si306 and pro-Si306 or their combination with Baf A1. Nuclei were counterstained with Hoechst 33342. Scale bar = 100 μm. (**d**) Histograms show LC3-positive puncta number and anti-p62 fluorescence intensity (arbitrary units) in treated U87-TxR cells normalized to control U87-TxR cells and expressed as percentages. Values are presented as mean ± SEM (*n* = 4). Statistically significant difference between treated and control groups is shown as * (*p* < 0.05), ** (*p* < 0.01), and *** (*p* < 0.001). Statistically significant difference between single STKI treatments and combined treatment with Baf A1 is shown as ## (*p* < 0.01).

To further confirm the observed effects of STKIs on autophagy, we then assessed the cellular distribution and expression of key autophagy markers LC3 and p62 after 48 h treatment with Si306 and pro-Si306 (Figure 4). The accumulation of LC3-positive puncta at 48 h after autophagic flux blockade by Baf A1 demonstrated that autophagy is active at basal levels in both parental and MDR glioblastoma cells. Immunostaining for LC3 also revealed that autophagy was significantly increased upon STKI treatments (Figure 4a,c). Si306 and its prodrug showed similar efficacy in triggering the accumulation of LC3-positive puncta in both cell lines. Namely, the number of autophagosomes in U87 and U87-TxR cells increased over two-fold following the treatments with Si306 and pro-Si306 (Figure 4b,d). The inhibition of lysosomal function by Baf A1 resulted in additional accumulation of LC3-positive puncta in STKI-treated cells, strongly indicating induction of autophagy by Si306 and its prodrug (Figure 4a,c). Baf A1 combination with STKIs increased the number of autophagosomes approximately 50% (Si306) and 65% (pro-Si306) in U87 cells (Figure 4b) and over 70% in U87-TxR cells (Figure 4d) compared with single STKI treatments. The accumulation of autophagosomes after co-treatments with STKIs and Baf A1 was also accompanied by significantly increased levels of autophagosome substrate p62 due to autophagic flux blockade during synergistic combination treatments (Figure 4a–d).

**Figure 5:**
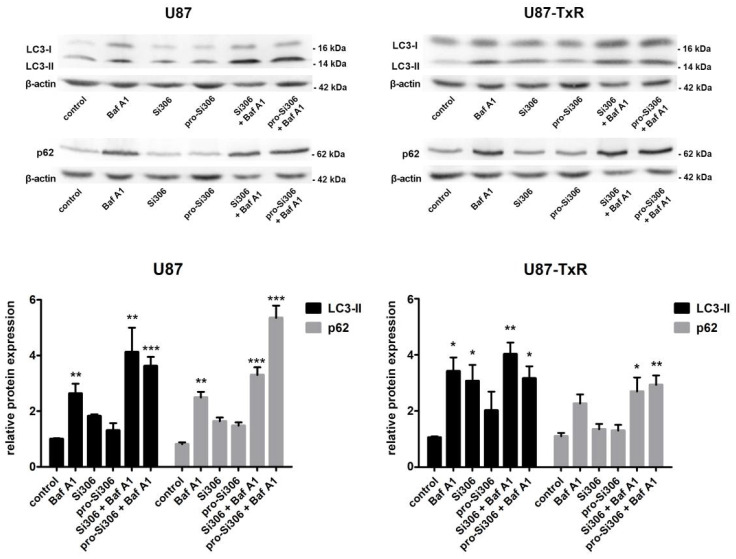
The effect of Si306 and pro-Si306 on the expression of autophagy markers LC3 and p62 in glioblastoma cells in single and combined treatments with Baf A1. Representative Western blot images of LC3 and p62 protein expression in U87 and U87-TxR cells after 48 h treatment with Si306 and pro-Si306 or their combination with Baf A1 are shown. Histograms represent LC3-II and p62 protein levels normalized to β-actin. Values are presented as mean ± SEM (*n* = 3). Statistically significant difference between treated and control groups is shown as * (*p* < 0.05), ** (*p* < 0.01), and *** (*p* < 0.001).

The effect of STKIs on autophagy was also evaluated by immunoblotting after 48 h (Figure 5). Our findings showed that the addition of Baf A1 increased the levels of autophagosome-bound LC3-II 4-fold in U87 cells treated with Si306, and 3.5-fold in cells treated with pro-Si306, compared with the control. A similar trend was observed in the U87-TxR cell line, where Baf A1 addition increased the levels of LC3-II 4-fold in Si306-treated cells and three-fold in prodrug-treated cells, strongly indicating the induction of autophagy by STKIs. A significant increase in p62 levels was also detected in parental and MDR glioblastoma cells treated with STKIs and Baf A1, initiated by inhibition of autophagic flux. Specifically, in combined treatments with STKIs and Baf A1, p62 levels were higher over 3-fold (Si306) and 5-fold (pro-Si306) in U87 cells, and over 2.5-fold in U87-TxR cells, compared with the control (Figure 5).

### 3.4. Bafilomycin A1 Enhances the Antiproliferative Effects of Si306 and Pro-Si306 in Glioblastoma Cells

**Figure 6:**
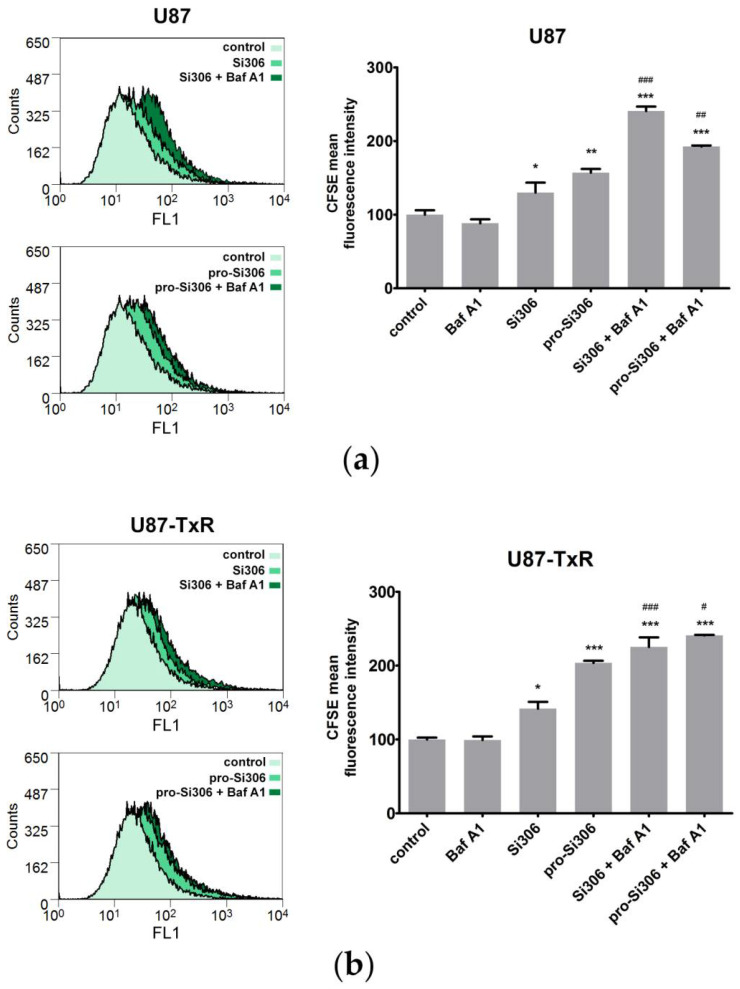
Baf A1 enhances the antiproliferative effects of Si306 and pro-Si306 in glioblastoma cells. (**a**) Antiproliferative activity in U87 cells was evaluated after 48 h in single treatments with Si306 and pro-Si306 or combined treatments with Baf A1. Representative flow-cytometric profiles of CFSE-labeled U87 cells are shown on the left panel. The histogram on the right panel shows CFSE fluorescence intensity (arbitrary units) in treated U87 cells relative to control U87 cells. Values are presented as mean ± SEM (*n* = 3). Statistically significant difference between treated and control groups is shown as * (*p* < 0.05), ** (*p* < 0.01), and *** (*p* < 0.001). Statistically significant difference between single STKI treatment and combined treatment with Baf A1 is shown as ## (*p* < 0.01) and ### (*p* < 0.001). (**b**) Antiproliferative activity in U87-TxR cells was evaluated after 48 h in single treatments with Si306 and pro-Si306 or combined treatments with Baf A1. Representative flow-cytometric profiles of CFSE-labeled U87-TxR cells are shown on the left panel. The histogram on the right panel shows the CFSE fluorescence intensity (arbitrary units) in treated U87-TxR cells relative to the control U87-TxR cells. Values are presented as mean ± SEM (*n* = 3). Statistically significant difference between treated and control groups is shown as * (*p* < 0.05) and *** (*p* < 0.001). Statistically significant difference between single STKI treatment and combined treatment with Baf A1 is shown as # (*p* < 0.05) and ### (*p* < 0.001).

We then used flow cytometry to determine whether the inhibition of cell proliferation contributed to the observed synergism between STKIs and Baf A1. Both STKIs showed the ability to inhibit the proliferation of glioblastoma cell lines, as revealed by CFSE staining (Figure 6). Treatment with 10 µM Si306 resulted in approximately 30% higher CFSE signal in U87 cells when compared with the control and 40% stronger signal in U87-TxR cells. Pro-Si306 treatment resulted in approximately 60% stronger CFSE fluorescence in U87 cells and two-fold stronger fluorescence in U87-TxR cells. While 20 nM Baf A1 did not significantly affect cell proliferation, the combination of Baf A1 and 10 µM STKIs resulted in a significantly stronger antiproliferative effect compared with STKIs alone (Figure 6). Simultaneous treatment with Si306 and Baf A1 resulted in over 2.4-fold and 2.2-fold stronger CFSE signals in U87 and U87-TxR cells, respectively, when compared with the control cells. Pro-Si306 in combination with Baf A1 resulted in 1.9-fold and 2.4-fold stronger fluorescence in U87 and U87-TxR cells, respectively (Figure 6).

### 3.5. Si306 and Pro-Si306 in Combination with Bafilomycin A1 Induce Necrotic Cell Death in Glioblastoma Cells

**Figure 7:**
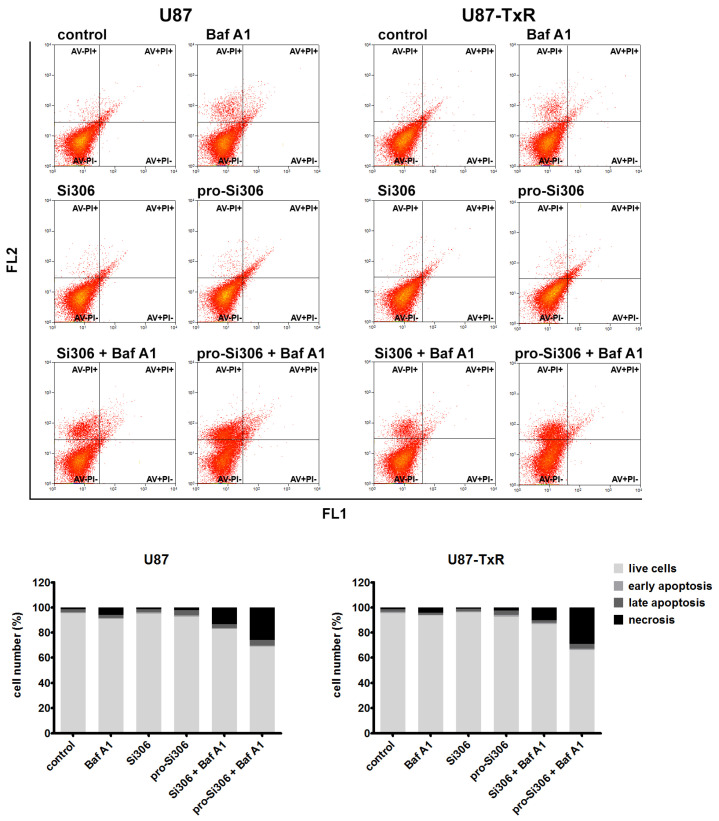
Si306 and pro-Si306 in combination with Baf A1 induce necrotic cell death in glioblastoma cells. AV/PI staining was used to assess cell death in U87 and U87-TxR cells following 48 h single treatments with Si306 and pro-Si306 or combined treatments with Baf A1. Representative flow-cytometric profiles of AV/PI-labeled cells are shown for each condition. Histograms in the bottom panel show the percentage of viable (AV− PI−), early apoptotic (AV+ PI−), late apoptotic (AV+ PI+), and necrotic (AV− PI+) cells after treatments.

To assess whether the induction of cell death accompanied inhibition of cell proliferation and the observed synergism between STKIs and Baf A1, glioblastoma cells were analyzed by flow cytometry using AV/PI staining. Treatment with 10 µM Si306 or pro-Si306 did not induce substantial cell death in either U87 or U87-TxR cells, nor did treatment with 20 nM Baf A1 (Figure 7). However, the addition of Baf A1 to STKI treatments considerably improved their efficacy and increased the number of dead cells, especially with prodrug treatments. Flow-cytometric analysis of U87 cells revealed that after the simultaneous application of Si306 and Baf A1, cells mostly underwent necrosis (over 13% of cells), while the percentage of cells in late apoptosis was 3% (Figure 7). After co-treatment with pro-Si306 and Baf A1, 26% of U87 cells were necrotic and 4% were in late apoptosis. Similarly, co-application of Si306 and Baf A1 in U87-TxR cells resulted in 10% of necrotic cells and 2% of cells in late apoptosis. Co-treatment with pro-Si306 and Baf A1 resulted in 29% of MDR cells in necrosis and nearly 4% in late apoptosis (Figure 7).

## 4. Discussion

In this study, we investigated the effect of Si306 and its prodrug pro-Si306 on autophagy and the potential of autophagy modulation to sensitize glioblastoma cells toward these compounds. The properties of tested pyrazolo[3,4-*d*]pyrimidine derivatives and Src tyrosine kinase inhibitors were evaluated in the human glioblastoma U87 cell line and its multidrug-resistant counterpart U87-TxR. The presented findings are in addition to the effects of investigated compounds on glioblastoma cells reported in our earlier studies [18,19,20].

We previously showed that both glioblastoma cell lines used in this study, U87 and U87-TxR, are more resistant to apoptosis compared with other cancer cells [30,31]. However, MDR U87-TxR cells are characterized by different molecular and cytogenetic characteristics that further contribute to their resistance to different therapeutics compared with the parental U87 cell line [32]. Specifically, features such as overexpression of P-glycoprotein and inactivation of p53 are key factors contributing to the multidrug-resistant phenotype of U87-TxR cells. In this study, we observed higher basal LC3-II levels as well as more abundant autophagosomes in U87 compared with MDR cells. However, after the lysosomal inhibition by Baf A1, both cell lines accumulated a comparable number of autophagosomes. This may be an indication of an increased rate of autophagosome formation and degradation under basal conditions in MDR cells compared with the parental cell line. Given that efficient autophagy is one of the main mechanisms supporting the development of drug resistance in cancer cells, it is likely that autophagy represents an additional feature by which U87-TxR cells sustain their MDR phenotype.

In this study, we showed the potential of Src tyrosine kinase inhibitors Si306 and pro-Si306 to induce autophagy in glioblastoma cells. It is now well demonstrated from a variety of studies that different types of cancer cells exposed to tyrosine kinase inhibitors can engage autophagy in response to chemical insults to alleviate cellular stress [1,2,4,33,34]. SFK inhibitors such as PP2 and saracatinib have been shown to effectively induce autophagy in prostate cancer cells, while dasatinib and imatinib induced autophagy in glioblastoma and chronic myelogenous leukemia, respectively [4]). Dasatinib was also responsible for the potent induction of autophagy in the non-small cell lung carcinoma cell lines [1]. Other tyrosine kinase inhibitors, such as ibrutinib, gefitinib, erlotinib, lapatinib, and neratinib, also induced autophagy in different cancer types [2,34].

An additive effect in LC3-II levels after a treatment in the presence of a lysosomal inhibitor is a known indicator of increased autophagic flux in cells [26]. An increase in LC3-II levels after treatment in combination with Baf A1 compared with Baf A1 alone may also point to the increased synthesis of autophagy-related membranes [26]. We observed a prominent increase in LC3-II levels in cells treated with Si306 and pro-Si306 in the presence of Baf A1, which is a strong indication of autophagy induction by STKIs, further supported by the accumulation of autophagosomes in the cytoplasm. In addition, an increase in autophagosome-bound mRFP-LC3 was observed in transfected U87 and U87-TxR cells after treatment with Si306 and pro-Si306, which strongly indicated elevated levels of autophagy. Prior reports have shown that SFK inhibitors can induce autophagy through mTOR inhibition [4,35,36]. The PI3K/Akt/mTOR axis is the main regulatory pathway that suppresses autophagy and is regulated by Src. We have previously demonstrated that Si306 and pro-Si306 inhibit Src tyrosine kinase activity in U87 and U87-TxR glioblastoma cells, along with the activity of upstream and downstream members of the Src signaling pathway, including components of the PI3K/Akt/mTOR axis [19]. The effect of STKIs on this pathway is likely to be one of the key drivers for the induction of autophagy by these inhibitors. In addition to their ability to inhibit the Src signaling pathway, Si306 and pro-Si306 are also inhibitors of P-glycoprotein, giving them the potential to overcome the MDR phenotype of U87-TxR cells [20]. It is important to point out that although the induction of autophagy may favor drug resistance, it does not override the P-glycoprotein inhibitory effect of these agents.

Existing research recognizes the critical role of autophagy in drug resistance after targeted therapy [25]. This has consequently prompted the application of autophagy inhibitors to revert the resistance and increase the efficacy of targeted therapeutics in vitro and in vivo. Combination therapy with tyrosine kinase inhibitors and well-tested autophagy inhibitors has helped reverse induced drug resistance in various cancer types including breast, colorectal, liver, lung, prostate cancer, melanoma, and hematological malignancies [25]. The suppression of autophagy using chloroquine (CQ), a blocking agent of lysosomal metabolism and autophagic flux [37], was found to reverse resistance to tyrosine kinase inhibitors such as erlotinib [38], ponatinib [10], saracatinib, and PP2 [4]. Enhanced cytotoxicity of certain drugs after late-stage autophagy inhibition has also been demonstrated in glioblastoma. For instance, treatment with CQ triggered robust apoptosis in glioblastoma cell lines after arsenic trioxide addition [39]. Similarly, Baf A1 was shown to increase the cytotoxic effects of temozolomide in glioblastoma [40]. When it comes to tyrosine kinase inhibitors, a study has shown that autophagy inhibition by CQ enhanced the pro-apoptotic effects of vandetanib in U251 and U87 glioblastoma cells [41]. Similarly, a combination of CQ and NVP-BEZ235, an inhibitor of the PI3K/mTOR pathway, led to apoptosis in several glioblastoma cell lines in vitro and a growth reduction in glioblastoma xenografts in vivo [42]. Pharmacological inhibition of autophagy with CQ also helped overcome resistance to the BRAF inhibitor vemurafenib in glioblastoma cells [43]. However, the modulation of autophagy to kill glioblastoma cells has involved the use of both inhibitors and inducers of autophagy [44], so the function of autophagy in glioblastoma resistance remains unclear.

Many efforts have been made in clinical trials to overcome drug resistance but without significant success. The use of CQ was unfortunately accompanied by toxicity, which limited its widespread use in oncology [45]. Similarly to CQ, Baf A1 also demonstrated considerable anticancer potential in vitro [46,47], but its use in the clinic had limitations due to toxicity [48]. Still, the inhibition of autophagy by Baf A1 in combination with different anticancer drugs, including targeted therapeutics, is considered a promising therapeutic approach. In this study, we showed that suppression of autophagy with Baf A1 enhanced the efficacy of Si306 and its prodrug in glioblastoma cells. Our results suggest that Si306 and pro-Si306 do not induce significant apoptosis in U87 and MDR U87-TxR cells but rather have a pronounced antiproliferative effect. This is likely due to the inhibitory effect of STKIs on Src signaling pathway components that regulate cell growth and proliferation, such as ERK, as reported in our previous study [19]. However, a combination of Baf A1 and STKIs had a strong synergistic effect in U87 and U87-TxR glioblastoma cells, which was evident through the effects on cellular metabolic activity, cell proliferation, and cell death induction. During combination treatments, autophagic flux was disrupted as shown by lysosomal deacidification, autophagosome accumulation, and increased levels of p62, an LC3-binding protein degraded by autophagy [49]. Taken together, these findings indicate that STKI-induced autophagy functions as an adaptive survival mechanism to overcome the anticancer effects of STKIs on glioblastoma cells.

While autophagy plays a predominantly suppressive role in necrotic cell death, inhibition of autophagy in apoptosis-impaired cells can promote necrotic cell death in vitro and in vivo [50,51]. Due to their defective apoptosis, the modulation of autophagy in glioblastoma may be an alternative way to trigger cell death. The inhibition of autophagy not only improved the antiproliferative potential of Si306 and pro-Si306 but also triggered substantial necrosis in U87 and U87-TxR cells. This result is consistent with our recent study, in which we found that STKIs triggered necrotic cell death in patient-derived glioblastoma cells [18]. It is important to highlight that STKI treatments caused significant necrosis in primary cultures without inhibition of autophagy, which is not surprising given that primary glioblastoma cells showed much greater sensitivity to Si306 and pro-Si306 compared with the U87 and U87-TxR cell lines [18,19].

The existence of the necrotic core in glioblastoma may promote cell invasion into the healthy surrounding tissue [52]. However, necrosis present in the glioblastoma tumor core should be distinguished from necrosis resulting from therapy. The former is caused by the lack of available oxygen, while the latter is caused by the action of the drug and can involve different mechanisms. Treatment-induced necrosis may eliminate potentially invading cells located closer to functional blood vessels, not only cells present in the tumor core, which would make drug-induced necrosis in glioblastoma a favorable outcome.

## 5. Conclusions

In summary, our results suggest that Si306 and pro-Si306 induce autophagy and that the use of autophagy inhibitors may increase the cytotoxicity of these pyrazolo[3,4-*d*]pyrimidine derivatives. The combination of Si306 or its prodrug with Baf A1 may help overcome MDR and lessen side effects by reducing drug doses due to the synergistic nature of their interaction, giving these Src tyrosine kinase inhibitors good translational potential. Considering their features as blood–brain barrier-penetrating drugs, Si306 and pro-Si306 could be good candidates for targeted therapy of glioblastoma. Therefore, the Src tyrosine kinase inhibitors’ combination treatment strategy explored in this study undoubtedly warrants further investigation.

## Data Availability

The data presented in this study are available from the corresponding author upon reasonable request.

## Supplementary Materials

The following supporting information can be downloaded at: https://www.mdpi.com/article/10.3390/life12101503/s1, Figure S1: Sensitivity of U87 and U87-TxR cell lines to bafilomycin A1 after 48 h treatment; Figure S2: Synergistic type of interaction between Src tyrosine kinase inhibitors and bafilomycin A1 in glioblastoma cells.

## References

[1] Tanaka H., Hino H., Moriya S., Kazama H., Miyazaki M., Takano N., Hiramoto M., Tsukahara K., Miyazawa K. (2020). Comparison of autophagy inducibility in various tyrosine kinase inhibitors and their enhanced cytotoxicity via inhibition of autophagy in cancer cells in combined treatment with azithromycin. Biochem. Biophys. Rep..

[2] Silva V.R., Neves S.P., Santos L.S., Dias R.B., Bezerra D.P. (2020). Challenges and Therapeutic Opportunities of Autophagy in Cancer Therapy. Cancers.

[3] Chang H., Zou Z. (2020). Targeting autophagy to overcome drug resistance: Further developments. J. Hematol. Oncol..

[4] Wu Z., Chang P.C., Yang J.C., Chu C.Y., Wang L.Y., Chen N.T., Ma A.H., Desai S.J., Lo S.H., Evans C.P. (2010). Autophagy Blockade Sensitizes Prostate Cancer Cells towards Src Family Kinase Inhibitors. Genes Cancer.

[5] Olivier C., Oliver L., Lalier L., Vallette F.M. (2020). Drug Resistance in Glioblastoma: The Two Faces of Oxidative Stress. Front. Mol. Biosci..

[6] Khan I., Baig M.H., Mahfooz S., Rahim M., Karacam B., Elbasan E.B., Ulasov I., Dong J.J., Hatiboglu M.A. (2021). Deciphering the Role of Autophagy in Treatment of Resistance Mechanisms in Glioblastoma. Int. J. Mol. Sci..

[7] Ou A., Yung W.K.A., Majd N. (2020). Molecular Mechanisms of Treatment Resistance in Glioblastoma. Int. J. Mol. Sci..

[8] Jiang P., Mukthavaram R., Chao Y., Bharati I.S., Fogal V., Pastorino S., Cong X., Nomura N., Gallagher M., Abbasi T. (2014). Novel anti-glioblastoma agents and therapeutic combinations identified from a collection of FDA approved drugs. J. Transl. Med..

[9] Li X., Zhou Y., Li Y., Yang L., Ma Y., Peng X., Yang S., Liu J., Li H. (2019). Autophagy: A novel mechanism of chemoresistance in cancers. Biomed. Pharmacother..

[10] Corallo D., Pastorino F., Pantile M., Mariotto E., Caicci F., Viola G., Ponzoni M., Tonini G.P., Aveic S. (2020). Autophagic flux inhibition enhances cytotoxicity of the receptor tyrosine kinase inhibitor ponatinib. J. Exp. Clin. Cancer Res. CR.

[11] Onorati A.V., Dyczynski M., Ojha R., Amaravadi R.K. (2018). Targeting autophagy in cancer. Cancer.

[12] Chude C.I., Amaravadi R.K. (2017). Targeting Autophagy in Cancer: Update on Clinical Trials and Novel Inhibitors. Int. J. Mol. Sci..

[13] Litwack G. (2018). Enzymes.

[14] Ahluwalia M.S., de Groot J., Liu W.M., Gladson C.L. (2010). Targeting SRC in glioblastoma tumors and brain metastases: Rationale and preclinical studies. Cancer Lett..

[15] Pottier C., Fresnais M., Gilon M., Jerusalem G., Longuespee R., Sounni N.E. (2020). Tyrosine Kinase Inhibitors in Cancer: Breakthrough and Challenges of Targeted Therapy. Cancers.

[16] Schenone S., Radi M., Musumeci F., Brullo C., Botta M. (2014). Biologically driven synthesis of pyrazolo[3,4-d]pyrimidines as protein kinase inhibitors: An old scaffold as a new tool for medicinal chemistry and chemical biology studies. Chem. Rev..

[17] Calgani A., Vignaroli G., Zamperini C., Coniglio F., Festuccia C., Di Cesare E., Gravina G.L., Mattei C., Vitale F., Schenone S. (2016). Suppression of SRC Signaling Is Effective in Reducing Synergy between Glioblastoma and Stromal Cells. Mol. Cancer Ther..

[18] Kostic A., Jovanovic Stojanov S., Podolski-Renic A., Nesovic M., Dragoj M., Nikolic I., Tasic G., Schenone S., Pesic M., Dinic J. (2021). Pyrazolo[3,4-d]pyrimidine Tyrosine Kinase Inhibitors Induce Oxidative Stress in Patient-Derived Glioblastoma Cells. Brain Sci..

[19] Nesovic M., Divac Rankov A., Podolski-Renic A., Nikolic I., Tasic G., Mancini A., Schenone S., Pesic M., Dinic J. (2020). Src Inhibitors Pyrazolo[3,4-d]pyrimidines, Si306 and Pro-Si306, Inhibit Focal Adhesion Kinase and Suppress Human Glioblastoma Invasion In Vitro and In Vivo. Cancers.

[20] Fallacara A.L., Zamperini C., Podolski-Renic A., Dinic J., Stankovic T., Stepanovic M., Mancini A., Rango E., Iovenitti G., Molinari A. (2019). A New Strategy for Glioblastoma Treatment: In Vitro and In Vivo Preclinical Characterization of Si306, a Pyrazolo[3,4-d]Pyrimidine Dual Src/P-Glycoprotein Inhibitor. Cancers.

[21] Podolski-Renic A., Dinic J., Stankovic T., Tsakovska I., Pajeva I., Tuccinardi T., Botta L., Schenone S., Pesic M. (2021). New Therapeutic Strategy for Overcoming Multidrug Resistance in Cancer Cells with Pyrazolo[3,4-d]pyrimidine Tyrosine Kinase Inhibitors. Cancers.

[22] Zawilska J.B., Wojcieszak J., Olejniczak A.B. (2013). Prodrugs: A challenge for the drug development. Pharmacol. Rep..

[23] Vignaroli G., Iovenitti G., Zamperini C., Coniglio F., Calandro P., Molinari A., Fallacara A.L., Sartucci A., Calgani A., Colecchia D. (2017). Prodrugs of Pyrazolo[3,4-d]pyrimidines: From Library Synthesis to Evaluation as Potential Anticancer Agents in an Orthotopic Glioblastoma Model. J. Med. Chem..

[24] Podolski-Renic A., Andelkovic T., Bankovic J., Tanic N., Ruzdijic S., Pesic M. (2011). The role of paclitaxel in the development and treatment of multidrug resistant cancer cell lines. Biomed. Pharmacother..

[25] Mele L., Del Vecchio V., Liccardo D., Prisco C., Schwerdtfeger M., Robinson N., Desiderio V., Tirino V., Papaccio G., La Noce M. (2020). The role of autophagy in resistance to targeted therapies. Cancer Treat. Rev..

[26] Klionsky D.J., Abdel-Aziz A.K., Abdelfatah S., Abdellatif M., Abdoli A., Abel S., Abeliovich H., Abildgaard M.H., Abudu Y.P., Acevedo-Arozena A. (2021). Guidelines for the use and interpretation of assays for monitoring autophagy (4th edition)(1). Autophagy.

[27] Chou T.C., Talalay P. (1984). Quantitative analysis of dose-effect relationships: The combined effects of multiple drugs or enzyme inhibitors. Adv. Enzym. Regul..

[28] Kimura S., Noda T., Yoshimori T. (2007). Dissection of the Autophagosome Maturation Process by a Novel Reporter Protein, Tandem Fluorescent-Tagged LC3. Autophagy.

[29] Eng K.E., Panas M.D., Karlsson Hedestam G.B., McInerney G.M. (2010). A novel quantitative flow cytometry-based assay for autophagy. Autophagy.

[30] Dacevic M., Isakovic A., Podolski-Renic A., Isakovic A.M., Stankovic T., Milosevic Z., Rakic L., Ruzdijic S., Pesic M. (2013). Purine nucleoside analog--sulfinosine modulates diverse mechanisms of cancer progression in multi-drug resistant cancer cell lines. PLoS ONE.

[31] Podolski-Renic A., Bosze S., Dinic J., Kocsis L., Hudecz F., Csampai A., Pesic M. (2017). Ferrocene-cinchona hybrids with triazolyl-chalcone linkers act as pro-oxidants and sensitize human cancer cell lines to paclitaxel. Metallomics.

[32] Podolski-Renic A., Jadranin M., Stankovic T., Bankovic J., Stojkovic S., Chiourea M., Aljancic I., Vajs V., Tesevic V., Ruzdijic S. (2013). Molecular and cytogenetic changes in multi-drug resistant cancer cells and their influence on new compounds testing. Cancer Chemother. Pharmacol..

[33] Aveic S., Pantile M., Polo P., Sidarovich V., De Mariano M., Quattrone A., Longo L., Tonini G.P. (2018). Autophagy inhibition improves the cytotoxic effects of receptor tyrosine kinase inhibitors. Cancer Cell Int..

[34] Wang J., Liu X., Hong Y., Wang S., Chen P., Gu A., Guo X., Zhao P. (2017). Ibrutinib, a Bruton’s tyrosine kinase inhibitor, exhibits antitumoral activity and induces autophagy in glioblastoma. J. Exp. Clin. Cancer Res. CR.

[35] Pal R., Palmieri M., Chaudhury A., Klisch T.J., di Ronza A., Neilson J.R., Rodney G.G., Sardiello M. (2018). Src regulates amino acid-mediated mTORC1 activation by disrupting GATOR1-Rag GTPase interaction. Nat. Commun..

[36] Ahn J.H., Lee M. (2011). Suppression of autophagy sensitizes multidrug resistant cells towards Src tyrosine kinase specific inhibitor PP2. Cancer Lett..

[37] Yang Y.P., Hu L.F., Zheng H.F., Mao C.J., Hu W.D., Xiong K.P., Wang F., Liu C.F. (2013). Application and interpretation of current autophagy inhibitors and activators. Acta Pharmacol. Sin..

[38] Wang Z., Du T., Dong X., Li Z., Wu G., Zhang R. (2016). Autophagy inhibition facilitates erlotinib cytotoxicity in lung cancer cells through modulation of endoplasmic reticulum stress. Int. J. Oncol..

[39] Li C., Liu Y., Liu H., Zhang W., Shen C., Cho K., Chen X., Peng F., Bi Y., Hou X. (2015). Impact of autophagy inhibition at different stages on cytotoxic effect of autophagy inducer in glioblastoma cells. Cell. Physiol. Biochem. Int. J. Exp. Cell. Physiol. Biochem. Pharmacol..

[40] Kanzawa T., Germano I.M., Komata T., Ito H., Kondo Y., Kondo S. (2004). Role of autophagy in temozolomide-induced cytotoxicity for malignant glioma cells. Cell Death Differ..

[41] Shen J., Zheng H., Ruan J., Fang W., Li A., Tian G., Niu X., Luo S., Zhao P. (2013). Autophagy inhibition induces enhanced proapoptotic effects of ZD6474 in glioblastoma. Br. J. Cancer.

[42] Fan Q.W., Cheng C., Hackett C., Feldman M., Houseman B.T., Nicolaides T., Haas-Kogan D., James C.D., Oakes S.A., Debnath J. (2010). Akt and autophagy cooperate to promote survival of drug-resistant glioma. Sci. Signal..

[43] Mulcahy Levy J.M., Zahedi S., Griesinger A.M., Morin A., Davies K.D., Aisner D.L., Kleinschmidt-DeMasters B.K., Fitzwalter B.E., Goodall M.L., Thorburn J. (2017). Autophagy inhibition overcomes multiple mechanisms of resistance to BRAF inhibition in brain tumors. eLife.

[44] Escamilla-Ramirez A., Castillo-Rodriguez R.A., Zavala-Vega S., Jimenez-Farfan D., Anaya-Rubio I., Briseno E., Palencia G., Guevara P., Cruz-Salgado A., Sotelo J. (2020). Autophagy as a Potential Therapy for Malignant Glioma. Pharmaceuticals.

[45] Kamat S., Kumari M. (2021). Repurposing Chloroquine Against Multiple Diseases With Special Attention to SARS-CoV-2 and Associated Toxicity. Front. Pharmacol..

[46] Lu X., Chen L., Chen Y., Shao Q., Qin W. (2015). Bafilomycin A1 inhibits the growth and metastatic potential of the BEL-7402 liver cancer and HO-8910 ovarian cancer cell lines and induces alterations in their microRNA expression. Exp. Ther. Med..

[47] Whitton B., Okamoto H., Packham G., Crabb S.J. (2018). Vacuolar ATPase as a potential therapeutic target and mediator of treatment resistance in cancer. Cancer Med..

[48] Drose S., Altendorf K. (1997). Bafilomycins and concanamycins as inhibitors of V-ATPases and P-ATPases. J. Exp. Biol..

[49] Jiang P., Mizushima N. (2015). LC3- and p62-based biochemical methods for the analysis of autophagy progression in mammalian cells. Methods.

[50] Chaabane W., User S.D., El-Gazzah M., Jaksik R., Sajjadi E., Rzeszowska-Wolny J., Los M.J. (2013). Autophagy, apoptosis, mitoptosis and necrosis: Interdependence between those pathways and effects on cancer. Arch. Immunol. Ther. Exp..

[51] Degenhardt K., Mathew R., Beaudoin B., Bray K., Anderson D., Chen G., Mukherjee C., Shi Y., Gelinas C., Fan Y. (2006). Autophagy promotes tumor cell survival and restricts necrosis, inflammation, and tumorigenesis. Cancer Cell.

[52] Monteiro A.R., Hill R., Pilkington G.J., Madureira P.A. (2017). The Role of Hypoxia in Glioblastoma Invasion. Cells.

