# Supplementary material for: Autophagy Inhibition Enhances Anti-Glioblastoma Effects of Pyrazolo[3,4-d]pyrimidine Tyrosine Kinase Inhibitors
Source: Life (Basel). 2022 Sep 27;12(10):1503. doi: 10.3390/life12101503 (PMC9605466; doi:10.3390/life12101503)
Supplement: Supplementary file 1 [file life-12-01503-s001.zip › life-1881579-supplementary.pdf]

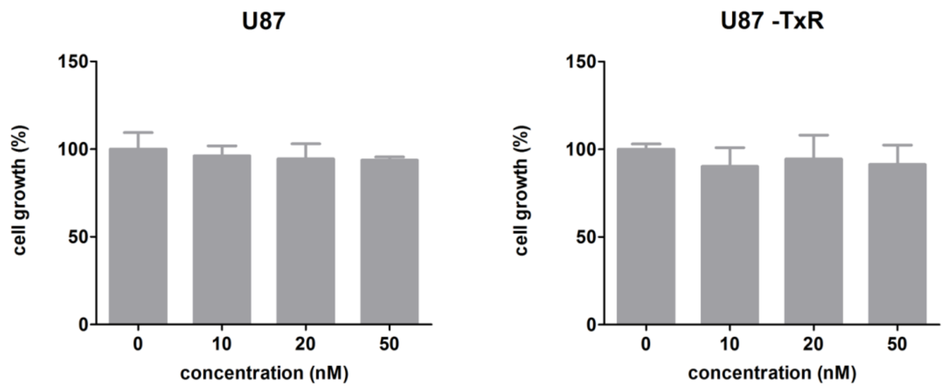

Figure S1. Sensitivity of U87 and U87-TxR cell lines to bafilomycin A1 after 48 h treatment. Cell growth inhibition was determined by MTT assay. Values are expressed as mean ± SEM (n = 3).

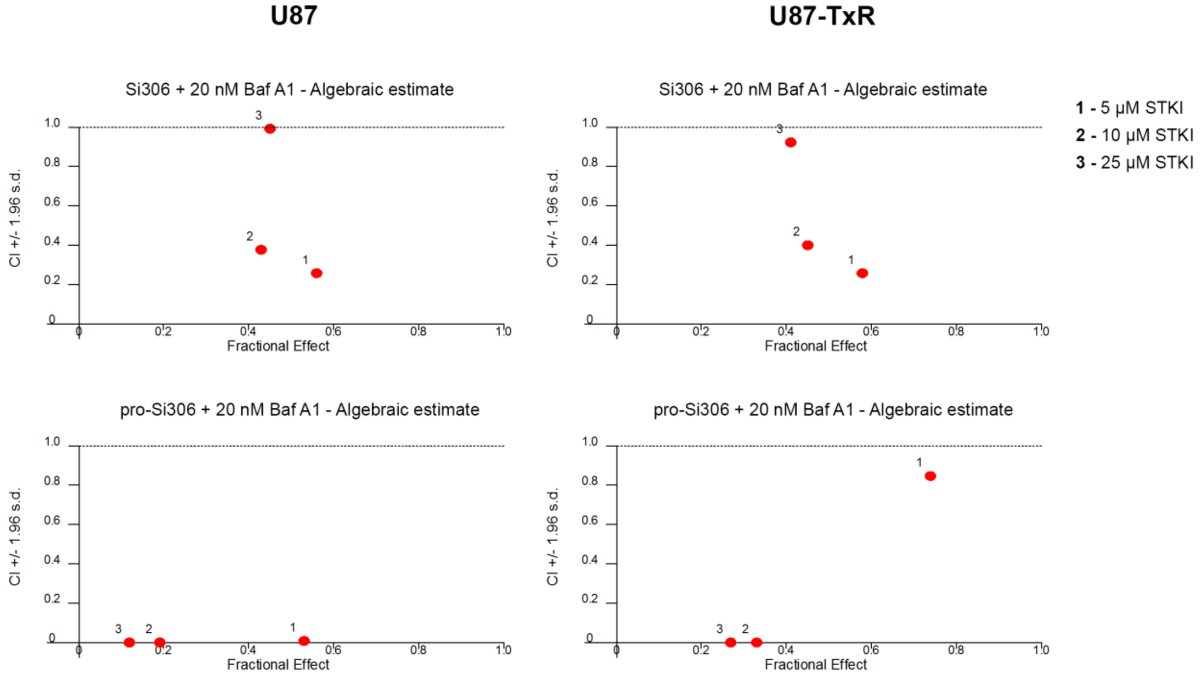

Figure S2. Synergistic type of interaction between Src tyrosine kinase inhibitors and bafilomycin A1 in glioblastoma cells. The interactions between multiple drug concentration combinations of Si306 and pro-Si306 with Baf A1 in U87 and U87-TxR cells were analyzed by CalcuSyn software and presented as an algebraic estimate for each combination. Combination Index (CI) values below 1 indicate synergistic interaction. CI values equal to 1 point to additive effect.
